# Nutrition and Selected Lifestyle Elements as a Tertiary Prevention in Colorectal Cancer Patients

**DOI:** 10.3390/nu16183129

**Published:** 2024-09-16

**Authors:** Kamil Michał Mąkosza, Małgorzata Muc-Wierzgoń, Sylwia Dzięgielewska-Gęsiak

**Affiliations:** 1 Doctoral School, Medical University of Silesia, 40-055 Katowice, Poland; 2 Department of Internal Diseases Propaedeutics and Emergency Medicine, Faculty of Public Health in Bytom, Medical University of Silesia, 40-055 Katowice, Poland; mwierzgon@sum.edu.pl (M.M.-W.); sgesiak@sum.edu.pl (S.D.-G.)

**Keywords:** nutrition, selected lifestyle elements, tertiary prevention, colorectal cancer patients

## Abstract

Background. Nutrition and lifestyle elements can significantly support the therapeutic process in colorectal cancer (*CRC*) patients, which is the basis for tertiary prevention. The study aimed to assess the nutritional strategies and lifestyle of *CRC* patients and to determine differences in these behaviors depending on gender and age. Methods. The study group included 202 *CRC* patients. The research was carried out in two hospitals and using the snowball method. The research tool was an original questionnaire. Data were processed in statistical programs. *p* < 0.05 was considered statistically significant. Results. Patients reported many behavioral–nutritional side effects. Half of them did not use a therapeutic diet (*n* = 101; 50.0%). The majority of patients declared that they ate three meals a day (57.4%). Fruits and vegetables were mainly eaten raw (69.3%). Almost a quarter of patients were not physically active at all (22.3%). Men chose to fry meat significantly more often than women (27.7% vs. 19.3%) (*p* = 0.003). The elderly consumed fast food significantly less often than middle-aged (88.5% vs. 72.3%) (*p* = 0.03). Conclusions. Patients showed both pro- and anti-health activities. The findings revealed several noteworthy disparities in dietary habits and lifestyle choices based on gender and age, indicating that these factors can significantly influence the health management of *CRC* patients. The patients’ behaviors should be constantly monitored and intensified, especially through regular consultations and educational meetings with an oncology dietitian for nutritional tertiary prevention of chronic disease.

## 1. Introduction

Malignant tumors are the second leading cause of death worldwide, with colorectal cancer (*CRC*) being highly prevalent in Europe [1,2]. The World Health Organization (*WHO*) reports that the incidence of *CRC* will continue to increase until 2040, indicating that the ratio of the number of newly diagnosed cases in 2040 will be 35% higher compared to the number of cases recorded in 2020 [3]. This upward trend in *CRC* cases may be linked to the changing demographic makeup of the population and the rising average life expectancy [1,4]. Researchers confirm the importance of diet and lifestyle in preventing and treating *CRC*. Moreover, they emphasize the need for public awareness of oncological prevention [5,6,7].

**Figure 1:**
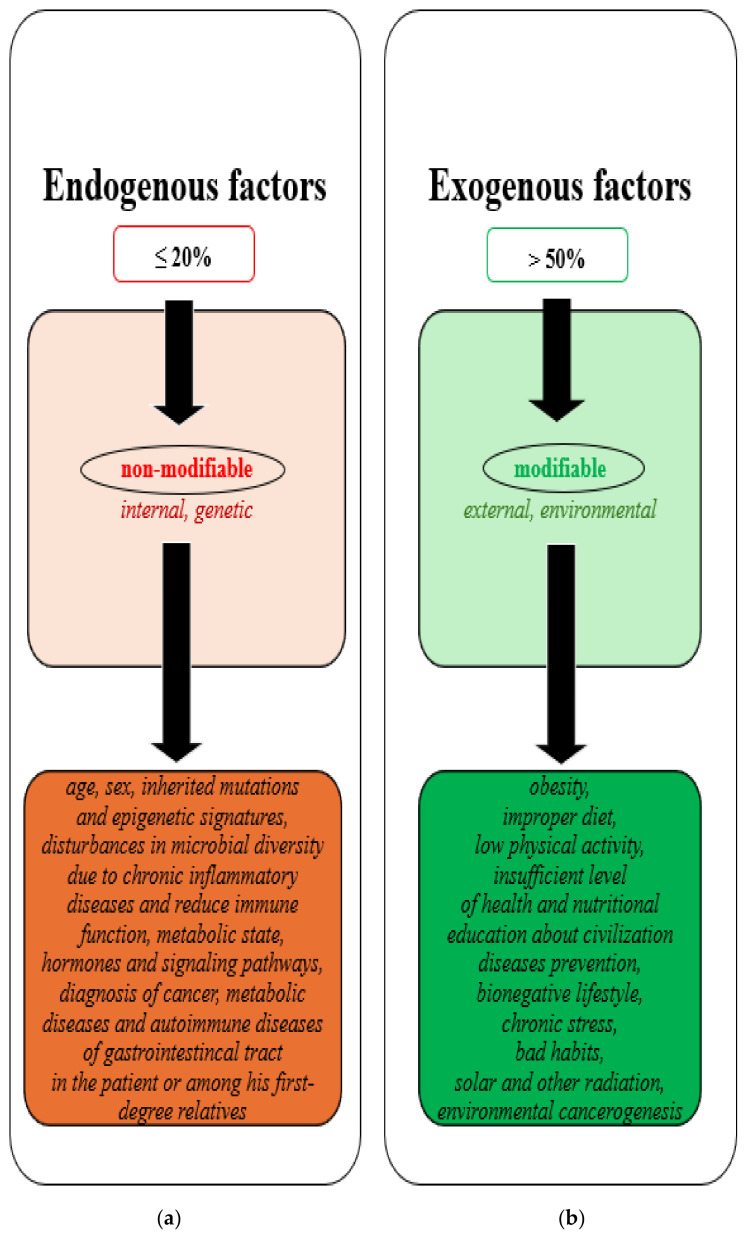
Factors responsible for the initiation of *CRC* and other types of cancer: (**a**) endogenous, non-modifiable factors; (**b**) exogenous, modifiable factors.

There are many factors responsible for the risk of initiating *CRC* and other types of cancer, such as breast cancer, lung cancer, and stomach cancer [8,9,10]. These include modifiable factors (responsible for at least 50% of the cause of cancers) and non-modifiable factors (responsible for at most 20% of the cause of cancers), as illustrated in Figure 1 [11,12].

Studies suggest that nutrition therapy and a biopositive lifestyle can improve the process of comprehensive oncological treatment, reduce the side effects of anticancer therapy and clinical symptoms, improve well-being with the overall quality of life, and may even lead to long-term remission of the disease through the applying of the rules of tertiary prevention [11,13,14]. 

Tertiary prevention seeks to reduce the impact of established disease by eliminating or reducing disability; minimizing suffering; and maximizing potential years of quality life, which refers to slowing disease progression and reducing complications stage disease [5]. In chronic conditions like cancer, the focus is on protecting the patient from adverse health effects such as malnutrition, cachexia, depression, and death [15,16]. Complex treatments including cancer therapy and supportive care are crucial for success and require multidisciplinary support: nutritional, psychological, social, and kinetic [17,18].

Nutrition therapy is crucial in the treatment of *CRC* patients and must be tailored to individual needs, considering factors such as gender, age, body mass index (*BMI*), activity level, tumor location, cancer stage and treatment type as well as comorbidities [19]. The diet should be easily digestible. The consistency of food should be adapted to the patient’s condition and his ability to eat, digest, and absorb food. Patients should eat regularly and their meals should be balanced and nutritious and consumed more frequently and in smaller portions. The diet should include complete sources of all macronutrients (proteins, fats, carbohydrates). Protein intake is important and can be supplemented with oral nutritional supplements [20]. Fats should come from sources like fatty fish and plant oils, while carbohydrates should be primarily complex [21,22]. Fiber intake should be tailored to individual tolerances and vegetables and fruits should be cooked to avoid irritation [23]. Meals should be attractive and nutritious to maintain appetite, with hydration being essential [24,25]. 

Physical activity is also recommended for *CRC* patients, in tertiary prevention, to improve well-being and overall quality of life [14]. It has been shown that that systematic, at least moderate physical activity can prevent it occurrence of the disease (primary prevention), prevent its recurrence, improve it parameters of oncological treatment, as well as improve the quality and comfort of the patient’s life struggling with cancer, which is important from the perspective of tertiary prevention [26]. Moderate physical exercise is about 150 min of exercise per week. It is worth taking care of to perform about 20–30 min of activity every day. The form, type, and intensity of physical exercise are individual and should be adequate to preferences and the patient’s kinetic and performance capabilities. Scientific research confirms that regular exercise improves the mental well-being and physical of the patient, eliminates the accompanying ailments, and has a positive effect on the results of many specialized studies, which is significant from the perspective of the role of tertiary cancer prevention [27]. It is crucial not to use stimulants such as cigarettes, alcohol, and other psychoactive substances. They may disturb the treatment process, worsen the patient’s clinical condition, intensify current side effects, initiate new ones, and also negatively affect the patient’s mental condition [28]. These actions are the complete opposite of tertiary prevention, so they should be avoided.

Anticancer treatments can lead to side effects like decreased appetite, taste changes, dry mouth, nausea, vomiting, diarrhea, and constipation, all of which can be managed through dietary adjustments [29]. The diet should be easily digestible and high in energy, but not too volumetric. The neutral smell of food is really essential. The importance of ginger is debated in the prevention of nausea and vomiting, however, current scientific evidence in this area is not yet consistent [30]. Additionally, ventilating rooms used for consumption may be helpful. Particularly recommended are jellies, puddings, legumes, soups, and mousses, as well as cocktails based on milk (in case of tolerance), vegetables, and fruit. During consumption, the patient should adopt an appropriate position—sitting or semi-sitting, to prevent the regurgitation of food. The therapeutic character of nutrition should be introduced, the so-called *BRAT* diet [31]. Patients suffering from constipation are advised to increase their level of physical activity, which will have a positive impact on intestinal transit [32]. Furthermore, it is recommended to consume 25–35 g of fiber in the daily diet, along with an adequate supply of fluids (at least 8–10 glasses a day). 

Thus, this study aimed to assess nutritional and lifestyle strategies in *CRC* patients, including identifying differences in the analyzed elements depending on gender and age among the study group.

## 2. Materials and Methods

### 2.1. Ethics

The study was conducted in accordance with the assumptions of the Declaration of Helsinki. Participation in the study was voluntary. Patients’ data were encoded by the pseudonymization procedure, which means that personal data is processed in such a way that it cannot be assigned to a specific data subject, without the use of an additional “key”. To ensure confidentiality and anonymity, all patient-identifying information was removed from the database before analysis. As a result, individual patients cannot be identified either in the article or the database. Additionally, since the data was anonymous and collected in a mandatory manner, obtaining informed consent from patients was not necessary. Patients were informed about the purpose of the study, its estimated duration, the right to withdraw from the study at any time, and the possibility to ask questions in case of any doubts.

### 2.2. Study Design

#### 2.2.1. Study Group and Methodology

The study group included 202 patients, 83 male and 119 female (age: 40–84 years; median age: 55 years), with colorectal adenocarcinoma (histopathological type adenocarcinoma) at four stages of clinical cancer according to the 9th classification of the Union for International Cancer Control/American Joint Committee on Cancer (UICC/AJCC). 

The patients were hospitalized in the Departments of Oncology of two Multispeciality Hospitals from the Silesia region (Poland), where the study was conducted. Additionally, the snowball method was used as a non-random sample selection to increase the size of the study group. The snowball method was used by reaching the study group in Silesia outside clinical facilities, after meeting the inclusion criteria.

Patients were recruited to the study from December 2023 to April 2024.

The inclusion criteria were as follows:

- Diagnosed and histopathologically confirmed *CRC*, regardless of the clinical stage;
- age: adults ≥18 years old;
- normal BMI (18.5–24.9 kg/m^2^);
- traditional oral nutrition;
- consent of the patient.

The exclusion criteria were as follows:

- Failure to meet any of the inclusion criteria, for example,
  - diagnosed other than *CRC* cancer;
  - age: <18 years old;
  - overweight or obese persons;
  - tube feeding or intravenous supplementation;
  - lack of patient consent.
- completion of a research tool inconsistently with the instructions;
- withdrawing from the study.

The research tool was an original, anonymous questionnaire related to the topic of the study. The development and validation of the authors’ questionnaire began with an initial draft list of the questionnaire items generated by the authors based on the literature. A pilot study had been previously conducted twice on a group of 30 subjects. The interval between the first and second surveys was 30 days, according to the arbitrary criteria used to assess the validity of the research tool. Based on the literature data, questions were asked about nutrition and lifestyle among *CRC* patients and evaluated in the questionnaire validation process. A question about the comparison of diet and lifestyle during and before cancer was removed from the original questionnaire due to an incorrect understanding of this question by patients. High or low levels were classified according to the median grouping of the dimension score.

The final questionnaire contained the following: tabular, open, semi-open, and closed, single-choice and multiple-choice questions. The first part of the questionnaire concerned the patient’s sociodemographic and medical data. Next, there was the proper part related to the research topic.

#### 2.2.2. Statistical Analyses

The study used the Statistica for Windows v. 13.3 software to collect and analyze the data, which is a statistical program designed for Windows. The χ^2^ test was employed to examine categorical variables, and the results were presented in terms of the number of cases and percentages. A statistical significance level was established, meaning that any *p*-value less than 0.05 was considered statistically significant. The comparative analyses whose results were statistically significant were presented. 

**Figure 2:**
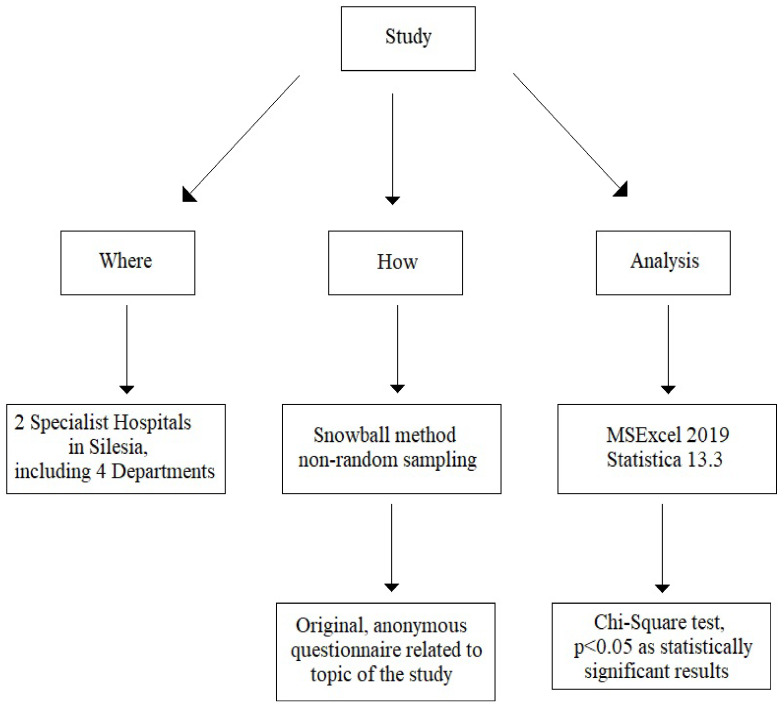
Study design.

The study design is presented in Figure 2.

## 3. Results

### 3.1. Characteristics of the Study Group

A total of 202 *CRC* patients were enrolled in the study. The study group was characterized depending on gender and age (Table 1). The majority of patients were women (58.9%) and middle-aged (69.3%).

### 3.2. Behavioral and Nutritional Problems of the Study Group

Patients indicated many side effects during the disease and the therapy (Table 2). The most common behavioral and nutritional problems among the study group were: xerostomia (67.3%), metallic taste (48.5%), and nausea (36.1%).

### 3.3. Nutritional Strategies among the Study Group

The half of patients did not have proper nutrition, i.e., an easily digestible diet. The majority of patients declared that they ate three meals a day (57.4%), and most often indicated eating snacks (61.4%), which were mainly a source of simple carbohydrates (33.2%). Fruits and vegetables were mainly eaten raw (69.3%). Dairy products were most often consumed several times a week (43.1%). Meat was most often consumed several times a week (77.2%), while fish was most often consumed several times a month (37.1%). Most patients did not consume fast food at all (77.2%), and almost half of the study group indicated boiling as a method of thermal processing of meat (45.1%) (Table 3). 

### 3.4. Lifestyle Strategies among the Study Group

The data related to hydration indicated that patients usually drank 1.5 L of fluids a day (50.0%), and chose sweetened drinks several times a month (33.7%). The preferred drink for most of the study group was natural mineral water (59.4%). The largest part of respondents declared that they did not usually consume ethanol (69.3%). The majority of patients indicated that they did not smoke cigarettes, but did before the diagnosis of the disease (53.5%). Almost a quarter of patients were not physically active at all (22.3%) (Table 4).

### 3.5. Nutritional and Lifestyle Strategies among the Study Group Depending on Gender

In the case of the process of thermal preparation of meat, women chose to roast significantly more often (8.4% vs. 0.0%), while men declared frying significantly more often (27.7% vs. 19.3%, *p* = 0.003). Women drank natural mineral water (61.3% vs. 56.6%) and tea (27.7% vs. 19.3%) significantly more often, while men were more likely to drink non-carbonated beverages (4.8% vs. 0.8%) and coffee (12.1% vs. 1.7%, *p* = 0.01) (Table 5).

### 3.6. Nutritional and Lifestyle Strategies among the Study Group Depending on Age

Both seniors and middle-aged people rarely consumed fast food, but the elderly did significantly less often—not at all (88.5% vs. 72.3%, *p* = 0.03). Elderly people were significantly more likely to have smoked before developing cancer (60.7% vs. 50.4%), while middle-aged people were significantly more likely to have smoked during their disease (6.4% vs. 0.0%, *p* = 0.04) (Table 6).

## 4. Discussion

The study group is predominantly female, which reflects a common trend in *CRC* demographics where women tend to frequent medical check-ups and are more likely to participate in studies. This inclination underscores the importance of addressing other lifestyle elements that play a pivotal role in the tertiary prevention of *CRC* [33]. In addition to nutrition, key lifestyle factors such as physical activity, stimulants, and various health behaviors and habits significantly influence both the course of treatment and the quality of life of *CRC* patients [34,35]. These elements are of great importance both in the incidence and survival in the context of *CRC* [36,37].

The original study showed that half of the patients did not follow the therapeutic type of the diet, however, the dietary choices did not differ between genders. Jackson et al. showed that following a diet with a limited supply of fatty acids helps reduce dyspeptic symptoms [38]. Some studies report that increased fat intake is a prognostic factor for poor outcomes in cancer survivors [39]. Mess et al. described that there is probably no clear clinical justification for absolutely minimizing the share of fatty acids in the diet, and only care should be taken to ensure their health-promoting type, source, and proper balance in the daily diet of *CRC* patients [40]. This is why an individualized diet is so important for cancer patients.

Half of the patients reported sufficient hydration (1.5 L/day)—the most commonly preferred liquid by respondents was natural mineral water, which was preferred more often by women than by men during cancer treatment. No current comparative data was found in the works in this respect other authors, but it is worth mentioning that during cancer, and especially during systemic treatment, adequate hydration is a very important factor, because side effects may occur deteriorated hydration homeostasis of the body, including dangerous dehydration [24]. What is more, promoting higher fluid intake and healthier drink choices, like more water instead of sugary drinks could be advantageous.

From the perspective of the role of nutrition in tertiary prevention, an adequate number of meals consumed during the day is important. The results of self-conducted research indicated that over half of patients eat only 3 meals a day, which may not be enough in this type of disease. Similar results reported by Dąbska et al., their respondents (53.0%) most frequently consumed 3–4 meals a day [41]. Karczmarek-Borowska et al. also presented such results—three meals (36.0%) and four meals (41.0%) [42]. However, taking into account the number of complications accompanying cancer patients, it will be much more beneficial to provide them with more meals a day, in smaller volumes, appropriately distributed over time—this may have a positive impact on the entire therapeutic process, including the nutritional status of patients. More frequently consumed, energy-rich meals (e.g., by fortifying the diet), but in smaller quantities, will not burden the gastrointestinal tract affected by the tumor [43].

The consequence of an incorrect number of meals consumed during the day may be the other problem—snacking between meals, especially those with anti-health effects. Our study presents that only a small percentage of patients admitted that they did not eat snacks between meals. Among the respondents who declared their consumption, the most frequently mentioned answer was products that were a source of simple carbohydrates—sweets and confectionery—and it is a type of unhealthy nutritional strategy. Fruit and vegetables as a snack were significantly less frequently preferred. Similar data were obtained by the already mentioned work of Karczmarek-Borowska et al., where 55.0% of people chose sweets as a snack, but much more often than in our research, vegetables or fruit were snacks (37.0%) [42]. Analyzes in the cohort study showed that subjects with a high intake of sugar and sweetened beverages, compared to subjects with a higher intake of fruit, vegetables, and whole grains, had poorer survival (higher risk of mortality, recurrence, and metastasis) [44]. Common nutritional errors require nutritional education as an integral element of primary, secondary, and tertiary cancer prevention.

Another aspect analyzed in the study group was the frequency of meat consumption and the thermal method of its preparation. Red and processed meat consumed in excessive amounts and too often, promote the process of cancer [45]. Instead of eating red meat, it is recommended to replace it with white meat, as well as to use products of plant origin that have no proven carcinogenic potential [46]. Our analysis showed that the largest percentage of respondents consume meat several times a week. The findings indicate significant differences in cooking methods between genders, with women prefer roasting meat and men prefer frying. This could point to differences in dietary fatty acid profiles and health implications. Di Maso et al. showed that 20.0% of cancer patients eat meat every day by frying it [47]. Pre-diagnosis processed meat dietary patterns are associated with a higher risk of cancer recurrence, metastasis, and mortality in *CRC* patients [48]. Studies have shown that patients who were on a diet containing more processed and red meat had increased recurrence rates and decreased disease-free survival rates [19]. According to the principles of any type of cancer prevention, it is worth constantly raising awareness about the harmful effects of excessive meat consumption and negative thermal methods, including frying, grilling, and smoking. These are the factors responsible for the uncontrolled progression of the disease, its recurrence, and even the initiation of another type of cancer in the future.

Research shows that increased fish consumption may be associated with a 33% reduction in the risk of *CRC*, which is an undoubted advantage in primary prevention [45]. In the case of fish consumption, the results obtained in the original research are unsatisfactory. A small part of respondents declared their consumption several times a month, and nearly a quarter of them indicated “once a month or less often”. In the study of Kiciak et al., fish consumption was defined as “once a week” [49]. Karczmarek-Borowska et al. noticed that 56.9% of the study group consumed fish more often in the past than during cancer [42]. The compared data prove that fish consumption is insufficient among people with cancer, which may have a negative impact on their nutritional status and the quantitative and qualitative diet. It is worth considering an alternative solution in the form of dietary supplements—omega-3 acids (*EPA* and *DHA*).

The study group in the original study frequently consumed dairy products, which is consistent with the assumptions of *CRC* prevention and treatment. Studies have shown that the calcium contained in dairy products reduces the risk of polyps and adenocarcinomas—a valuable element of primary prevention, and also reduces mortality from *CRC* by almost 30%, especially among the Western population—which is an essential factor in tertiary prevention [50,51,52].

Among the participants of our study, there was a problem with eating raw fruit in the majority of the study group. In the case of *CRC*, this form is not recommended due to frequent complications of the therapy used, therefore these products should be processed. The authors of self-conducted research did not find an analysis of the problem in the reports of other scientists, which encourages the expansion of scientific research among patients with gastrointestinal cancer in order to identify the form of consumption of specific products. Oncology patients should be educated about the possible consequences of consuming products in a specific form as nutritional tertiary prevention is crucial.

A potential protective effect on overall mortality in people diagnosed with *CRC* has been identified with a Mediterranean dietary pattern, although the results need to be confirmed in other large cohorts and studies [53]. Current research shows that the Mediterranean diet reduces the risk of *CRC* by 8–17% [54,55].

Anticancer therapy, in addition to degrading cancer cells, damages healthy cells, contributing to side effects. Self-conducted research showed that patients reported many different side effects: xerostomia, metallic taste, nausea, diarrhea, loss of appetite, constipation, vomiting, and others, including pain, reflux, and early feeling of satiety. Similar side effects have been described in the study by Snarska and Dolińska—nausea, loss of appetite, vomiting, constipation, diarrhea, and changes in the oral cavity [56]. Similar problems among the study group were described by Kapela et al.—nausea, pain, loss of appetite, diarrhea, constipation [57]. Based on the above-mentioned studies, it can be concluded that many side effects of cancer and oncological treatment are very common and therefore require targeted dietary management.

In the current research, a significant majority of participants were middle-aged. This is noteworthy since *CRC* incidence increases with age, particularly among individuals over 50, highlighting the importance of targeted interventions for this age group [58]. The consumption of fast food products in this study was negligible. What is more, middle-aged participants consumed fast food significantly more often than elderly *CRC* patients. This could correlate with health awareness and changes in taste or lifestyle preferences with age [59]. Other comparable findings were reported by other researchers—their research indicated that the intake of high-fat products prior to the disease was nearly two-thirds greater than what is currently observed among cancer patients. Research showed similar results [41,42]. The similarity of the results may be related to patients’ awareness of the harmful effects of highly processed food on the body, as well as the possibility of complicating therapeutic procedures in tertiary prevention of chronic disease.

The group of patients in the original study declared that they consumed ethanol quite rarely, which is consistent with the assumptions of primary, secondary, and tertiary cancer prevention. Donato et al. described a more frequent consumption of ethanol beverages than the respondents from self-conducted research [60]. Other research showed that the majority of respondents reported occasional alcohol consumption [49]. The term “occasionally” should be considered because the average respondent may interpret it completely differently, contrary to the researchers’ assumptions (e.g., for one respondent the term “occasionally” may mean “every few days”, and for another—“once a month or less often”). A meta-analysis of prospective studies showed a moderate positive association between alcohol abuse (>50 g/day) and *CRC*-related mortality [61]. It is impossible not to mention that the effects of alcohol are very toxic, especially in cancer patients [62,63]. In addition to damaging internal organs (mainly the liver), alcohol damages DNA, increases inflammation, and reacts with cytostatic drugs used during chemotherapy. This may intensify existing ailments, generate new ones, exacerbate an existing disease, or even initiate another, distant type of cancer, which negatively affects life expectancy [64].

The data on smoking show that elderly patients were more likely to have smoked before *CRC* diagnosis, but middle-aged patients were more inclined to smoke during their disease. Understanding these patterns could help tailor smoking cessation programs for different age *CRC* patient groups. Donato et al. published that smoking among respondents is much lower than the average smoking rate in the USA, where the study was carried out (approximately 4.5% vs. 18.0%) [60]. In the study by Zhu et al., it was reported that 72.6% of the respondents had ever smoked cigarettes (including current smokers), while the rest had never used this type of stimulant (27.4%) [65]. Kiciak et al. described that 69.0% of the study participants smoked cigarettes. The authors illustrated the exact quantitative scale of average cigarettes smoked per day. Most people declared smoking from 6 to 10 cigarettes a day, while a comparable number indicated 11 to 15 cigarettes smoked a day [49]. Amplitudes observed in the cited studies may be related, for example, to the dominant gender or level of education in the study group, to the predominance of a specific occupational or age group in which the average frequency of stimulant use may be significantly increased, or reduced. Smoking cigarettes during cancer significantly reduces the effectiveness of oncological treatment, thus shortening survival time. Moreover, it contributes to further unrestrained tumor growth and worsens the quality of life by intensifying current ailments. It may also cause metastases to distant organs and initiate the development of a new cancer. Smoking cigarettes and drinking alcohol even after cancer has been cured is undesirable, because people using stimulants may experience a recurrence of the disease and a serious exacerbation of the patient’s general condition [60].

Epidemiological data have shown that the risk of developing *CRC* is increased by 44% during a sedentary lifestyle [37]. A sedentary lifestyle leads to the development of obesity, which is associated with many metabolic disorders. These include inflammation, hyperinsulinemia, increased levels of sex hormones, and increased blood glucose levels. These are independent factors responsible for promoting the process of carcinogenesis and the development of *CRC* [66,67,68]. Declared, moderate levels of physical activity engaged by patients in self-conducted research were insufficient and terrifying. Prospective studies have demonstrated an association between greater physical activity and reduced mortality and disease recurrence in *CRC* without distant metastases [69]. In the study by Donato et al. on the health behaviors and lifestyle of people who survived *CRC* with and without being diagnosed with Lynch Syndrome, the level of physical exercise was also checked [60]. In this study, physical activity was declared for approximately 20.0% of men and approximately 28.0% of women. However, the frequency of implementation did not specify physical activity, its type, and intensity. Campbell et al. in their study showed that *CRC* patients who sat for more than 6 h/day post-diagnosis worsened the prognosis of the disease (all-cause mortality) by 27%. The cancer-related mortality even increased up to 63% in the study (2300 patients, observation period—16 years) [70]. A study conducted among stage III *CRC* patients showed that a biopositive and active lifestyle was associated with reduced cancer recurrence by 42% and reduced mortality by 57% [71]. In the study of Prokopowicz et al., related to the determinants of physical activity in women after cancer surgery breasts, it was shown that those examined were more likely to be physically active after mastectomy than before the diagnosis of cancer [72]. Women walked much more often (48.0% vs. 41.0%) and did sports gymnastics (64.0% vs. 35.0%) and swimming (47.0% vs. 35.0%) than in the period before diagnosis of the disease. Despite the fact that this study focused on a different type of cancer than in the original research, some recommendations related to a biopositive lifestyle, including regular physical activity, remain unchanged and are attributed to the primary, secondary, and tertiary prevention of chronic diseases, including cancer. Undoubtedly, the results obtained support the importance of physical activity in the therapeutic process of cancer, both in the context of improving the patient’s clinical condition, as well as his well-being and quality of life.

### 4.1. Strengths and Limitations of the Study

#### 4.1.1. Strengths

The study provides a thorough evaluation of nutritional habits, lifestyle choices, and their effects on *CRC* patients. What is more, the sample size is significant enough to detect patterns and differences in behaviors among different groups (age and gender). Moreover, such research may have practical implications as a comprehensive health education process for *CRC* patients. Based on the data obtained and frequently repeated unfavorable dietary and health behaviors in *CRC* patients, an educational meeting was conducted to improve the nutrition, lifestyle, and quality of life of the study group in the tertiary prevention of the disease.

#### 4.1.2. Limitations

The limitation of the study was its observational nature with retrospective elements. In the future, it is planned to expand the research by conducting prospective studies, analyzing more areas and factors, such as comprehensive nutritional assessment, including assessment of nutritional status with quantitative and qualitative assessment of diet, assessment of quality of life, and more detailed assessment of compliance with the principles of non-pharmacological tertiary prevention (lifestyle elements), as these will increase the reliability and effectiveness of the obtained data.

## 5. Conclusions

The study presents valuable insights into the dietary and lifestyle behaviors of *CRC* patients, highlighting the importance of tailored nutritional therapy as part of tertiary prevention.

Health-promoting behaviors in tertiary prevention of the study group include rare fast-food consumption, frequent consumption of dairy products, correct method of thermal processing of meat, moderate level of hydration, appropriate type of preferred drink, rarely drinking sweetened and alcoholic beverages, and not smoking cigarettes. These behaviors should be cultivated and the benefits associated with them should be emphasized to patients. Anti-health attitudes in tertiary prevention include not following an easily digestible diet, too few meals a day, eating vegetables and fruit in the wrong form, frequent consumption of simple carbohydrates, rare fish consumption, and insufficient level of physical activity. These attitudes require monitoring and improvement, mainly through ongoing advice and recommendations from an oncology dietitian.

The findings reveal several noteworthy disparities in dietary habits and lifestyle choices based on gender and age, indicating that these factors can significantly influence the health management of *CRC* patients. Moreover, the different dietary preferences exhibited by genders such as men tending toward frying meat and women opting for healthier cooking methods suggest that dietary interventions must be gender-sensitive to better cater to patient needs. Additionally, the elderly exhibited healthier fast food consumption patterns, indicating an awareness of dietary impacts on health that could be leveraged in educational interventions. 

The study also revealed a concerning lack of physical activity among *CRC* patients, which correlates with poorer health outcomes. Given the established link between physical activity and improved cancer prognosis, increasing awareness and encouraging regular exercise must become integral components of holistic cancer care within the framework of tertiary prevention.

Overall, the findings advocate for a personalized approach to dietary and lifestyle recommendations in *CRC* management, emphasizing the need for educational interventions to improve dietary practices and promote active lifestyles. Actions aimed at improving the nutrition and lifestyle of *CRC* patients should primarily include regular consultations and educational meetings with an oncology dietitian (especially during invasive treatment) to monitor the nutritional status and implement adequate, individual nutritional therapy. These efforts could enhance health outcomes and quality of life and support patients’ recovery and long-term survivorship in *CRC*.

## Figures and Tables

**Table 1:** General characteristics of the study group.

	*n*	%
Gender		
Woman	119	58.9
Man	83	41.1
Age		
Middle-aged	141	69.3
Elderly	61	30.2

**Table 2:** Behavioral and nutritional problems of the study group.

Kind of Problem	*n*	%
Xerostomia	136	67.3
Metallic taste	98	48.5
Nausea	73	36.1
Diarrhea	43	21.3
Loss of appetite	41	20.3
Constipation	22	10.9
Vomiting	17	8.4
Others (pain, reflux, early feeling of satiety)	18	8.9

**Table 3:** Nutritional strategies among the study group.

Nutritional Strategies	*n*	%
*Adherence to proper nutrition—an easily digestible diet*		
Yes	101	50.0
No	101	50.0
*Amount of meals consumed a day*		
<3	5	2.5
3	116	57.4
4–5	65	32.2
>5	16	7.9
*Eating snacks between meals*		
Yes	124	61.4
No	78	38.6
*Main types of snacks*		
Fruit	20	9.9
Vegetables	5	2.5
Sweets, confectionery	67	33.2
Salty products, fast food	19	9.4
Others	13	6.4
No consumption	78	38.6
*Most common form of consumption of fruit and vegetables*		
Cooked	32	15.8
Mixed	27	13.4
Raw	140	69.3
No consumption	3	1.5
*Frequency of dairy consumption*		
Every day	70	34.7
A few times a week	87	43.1
Once a week	11	5.4
Several times a month	21	10.4
Once a month or less often	7	3.5
At all	6	3.0
*Frequency of meat consumption*		
Every day	18	8.9
A few times a week	156	77.2
Once a week	22	10.9
Several times a month	2	1.0
Once a month or less often	1	0.5
At all	3	1.5
*Frequency of fish consumption*		
Every day	0	0.0
A few times a week	22	10.9
Once a week	42	20.8
Several times a month	75	37.1
Once a month or less often	48	23.8
At all	15	7.4
*Frequency of fast food consumption*		
Every day	0	0.0
A few times a week	0	0.0
Once a week	4	2.0
Several times a month	11	5.4
Once a month or less often	31	15.4
At all	156	77.2
*Thermal process of preparing meat*		
None (raw meat)	0	0.0
None (vegetarian diet)	3	1.5
Boiling	91	45.0
Frying	46	22.8
Roasting	10	5.0
Grilling	0	0.0
Stewing	52	25.7

**Table 4:** Lifestyle strategies among the study group.

Lifestyle Strategies	*n*	%
*Average level of daily hydration*		
≤0.5 L	5	2.5
About 1 L	36	17.8
About 1.5 L	101	50.0
About 2 L	38	18.8
>2 L	22	10.9
*Frequency of sweetened beverages drinking*		
Every day	7	3.5
A few times a week	31	15.3
Once a week	30	14.9
Several times a month	68	33.7
Once a month or less often	16	7.9
At all	50	24.8
*Type of beverage most often preferred*		
Sweetened, carbonated beverages	1	0.5
Non-carbonated beverages	5	2.5
Fruit compotes	3	1.5
Flavored water	12	5.9
Natural mineral water	120	59.4
Coffee	12	5.9
Tea	49	24.3
*Frequency of ethanol beverages drinking*		
Every day	0	0.0
A few times a week	0	0.0
Once a week	23	11.4
Several times a month	21	10.4
Once a month or less often	18	8.9
At all	140	69.3
*Cigarette smoking*		
Currently yes	18	8.9
Currently no, but before the disease yes	108	53.5
Currently no, but during the disease yes	9	4.5
Currently no and never before	67	33.2
*Frequency of physical activity*		
Every day	24	11.9
A few times a week	58	28.7
Once a week	43	21.3
Several times a month	28	13.9
Once a month or less often	4	2.0
At all	45	22.3

**Table 5:** Nutritional and lifestyle strategies among the study group depending on gender.

Nutritional and Lifestyle Strategies	Woman (*n* = 119) *n* (%)	Man (*n* = 83) *n* (%)	*p*
*Thermal process of preparing meat*			
None (raw meat)	0 (0.0)	0 (0.0)	
None (vegetarian diet)	3 (2.5)	0 (0.0)	
Boiling	55 (46.2)	36 (43.4)	
Frying	23 (19.3)	23 (27.7)	0.003
Roasting	10 (8.4)	0 (0.0)	
Grilling	0 (0.0)	0 (0.0)	
Stewing	28 (23.5)	24 (28.9)	
*Type of beverage most often preferred*			
Sweetened, carbonated beverages	0 (0.0)	1 (1.2)	
Non-carbonated beverages	1 (0.8)	4 (4.8)	
Fruit compotes	2 (1.7)	1 (1.2)	
Flavored water	8 (6.7)	4 (4.8)	0.01
Natural mineral water	73 (61.3)	47 (56.6)	
Coffee	2 (1.7)	10 (12.1)	
Tea	33 (27.7)	16 (19.3)	

**Table 6:** Nutritional and lifestyle strategies among the study group depending on age.

Nutritional and Lifestyle Strategies	Middle-Aged (*n* = 141) *n* (%)	Elderly (*n* = 61) *n* (%)	*p*
*Frequency of fast food consumption*			
Every day	0 (0.0)	0 (0.0)	
A few times a week	0 (0.0)	0 (0.0)	
Once a week	4 (2.8)	0 (0.0)	0.03
Several times a month	10 (7.1)	1 (1.6)	
Once a month or less often	25 (17.7)	6 (9.8)	
At all	102 (72.3)	54 (88.5)	
*Cigarette smoking*			
Currently yes	14 (9.9)	4 (6.6)	
Currently no, but before the disease yes	71 (50.4)	37 (60.7)	0.04
Currently no, but during the disease yes	9 (6.4)	0 (0.0)	
Currently no and never before	47 (33.3)	20 (32.8)	

## Data Availability

Data is contained within the article.
